# Biomarker Identification via Spatial Transcriptomics Profiling of Colorectal Cancer and Colorectal Cancer with Liver Metastasis Stem Cells

**DOI:** 10.3390/ijms262211045

**Published:** 2025-11-14

**Authors:** Minho Lee, Seoin Han, Hak Chun Kim, Yujun Jung, Jeong-An Gim, Chang-Jin Kim, Dongjun Jeong

**Affiliations:** 1 Department of Pathology, College of Medicine, Soonchunhyang University, 31 Soonchunhyang 6 gil, Dongnam-gu, Cheonan 31151, Chungcheongnam-do, Republic of Korea; leemho00@sch.ac.kr (M.L.); gkstjdls00@sch.ac.kr (S.H.); 2 Soonchunhyang Medical Science Research Institute, College of Medicine, Soonchunhyang University, 25 Bongjeong-ro, Dongnam-gu, Cheonan 31151, Chungcheongnam-do, Republic of Korea; khc5467@sch.ac.kr (H.C.K.); mascul@sch.ac.kr (Y.J.); 3 Department of Medical Science, Soonchunhyang University, 22 Suncheonhyang-ro, Asan-si 31538, Chungcheongnam-do, Republic of Korea; vitastar@sch.ac.kr; 4 Changwon Hanmaeum Hospital, Uichang-gu, Changwon-si 51139, Gyeongsangnam-do, Republic of Korea; mountain48@hanmail.net

**Keywords:** colorectal cancer, cancer stem cell, metastasis, digital spatial profiler, biomarkers, gene expression

## Abstract

Colorectal cancer (CRC) demonstrates favorable clinical outcomes when diagnosed at an early stage; however, the prognosis declines substantially following recurrence or distant metastasis. Increasing evidence indicates that cancer stem cells (CSCs) are pivotal contributors to tumor recurrence, metastatic dissemination, and therapeutic resistance. The present study aimed to identify CSC-associated biomarkers through spatial transcriptomic profiling of normal colonic mucosa, primary CRC, and liver metastatic tissues, and to evaluate their functional relevance in CRC progression. Spatial transcriptomic analysis revealed that CCN2 was preferentially enriched within CSC clusters of primary CRC tissues, whereas APOC2 was predominantly upregulated in liver-metastatic CSCs. Functional validation of CCN2 was performed by establishing CCN2-knockout HCT116 cell lines using the CRISPR-Cas9 system. Loss of CCN2 expression markedly attenuated cell proliferation, migration, invasion, and oxaliplatin resistance compared with control cells. Furthermore, immunohistochemical analysis of tissue microarrays demonstrated a significant positive correlation between CCN2 expression and CSC markers SOX2 and Nestin. Collectively, these findings suggest that CCN2 functions as a central regulator of stemness and malignant potential in CRC and may represent a promising therapeutic target to prevent recurrence and metastasis. Additional mechanistic studies are warranted to further elucidate the molecular pathways of CCN2 and to validate the role of APOC2 in liver-metastatic CRC stem cells.

## 1. Introduction

Globally, colorectal cancer (CRC) is the third most common malignancy, with approximately 1.9 million new cases diagnosed in 2022, and is a leading cause of cancer-related mortality, resulting in over 900,000 deaths annually [1]. According to global estimates, the incidence of CRC is 28.7 per 100,000 individuals in the United States and 10.0 per 100,000 in the United Kingdom, whereas South Korea reports the highest incidence worldwide at 45 per 100,000, with a continuous annual increase [1,2]. Survival outcomes for CRC are highly stage-dependent. Data from the National Cancer Information Center (2016–2020) indicate a 94.0% survival rate for early-stage disease (stages I–II), 82.5% for localized CRC, and only 20.0% for cases with distant metastasis [3,4]. More than 70% of CRC-related deaths are attributable to distant metastases, most frequently to the liver and peritoneum [5]. Surgical resection is feasible in fewer than 20% of metastatic CRC (mCRC) cases, with recurrence rates approaching 60%, underscoring the poor prognosis even after surgery [6,7]. Combination chemotherapy regimens, such as 5-fluorouracil (5-FU) with leucovorin (LV), FOLFOX, and FOLFIRI, remain the standard first-line treatments for mCRC; however, effective therapeutic strategies that significantly improve survival remain lacking [8,9].

Recurrence and therapeutic failure in CRC are largely attributed to a subpopulation of resistant cells, namely cancer stem cells (CSCs), which possess self-renewal and pluripotency properties [10,11]. CSCs play critical roles in CRC initiation, progression, recurrence, and metastasis, and are implicated in resistance to chemotherapy, radiotherapy, and targeted therapies [12]. Currently, CRC stem cell (CRCSC) identification relies on surface biomarkers such as CD133, CD166, CD44, CD24, CD138, EpCAM, ALDH1, CXCR4, and LGR5 [13,14,15]. Among these, CD44 is widely recognized as a master regulator of stemness, with established prognostic significance and therapeutic potential across multiple cancer types [16,17,18]. EpCAM^high^/CD44^+^ CRC cells exhibit stem cell-like properties and represent a major source of CSCs [13]. Nevertheless, markers that are both specific and exclusive to CRCSCs remain limited, highlighting the need to identify tumor-specific CSC markers that can distinguish them from normal stem cells.

Recent advances in spatial transcriptomics and proteomics have enabled high-resolution analysis of gene and protein expression in formalin-fixed paraffin-embedded (FFPE) tissues, facilitating simultaneous assessment of tumor and surrounding normal tissues [19]. The NanoString GeoMx Digital Spatial Profiler (DSP), a high-throughput spatial RNA quantification platform, allows for transcriptomic profiling from limited clinical samples. By quantifying target-specific oligonucleotide barcodes, this technology provides unprecedented insights into tumor immunology, tissue heterogeneity, and biomarker discovery [20].

In this study, we employed spatial transcriptomics to identify novel genes associated with CRC and mCRC by comparing stem cell populations in normal, primary tumor, and liver metastatic tissues. Our overarching goal was to uncover CRC stem cell-specific markers that may overcome therapeutic resistance and ultimately improve outcomes for patients with mCRC.

## 2. Results

### 2.1. RNA Expression in CSCs and Non-Stem Cells in Colorectal Primary Cancer

**Figure 1:**
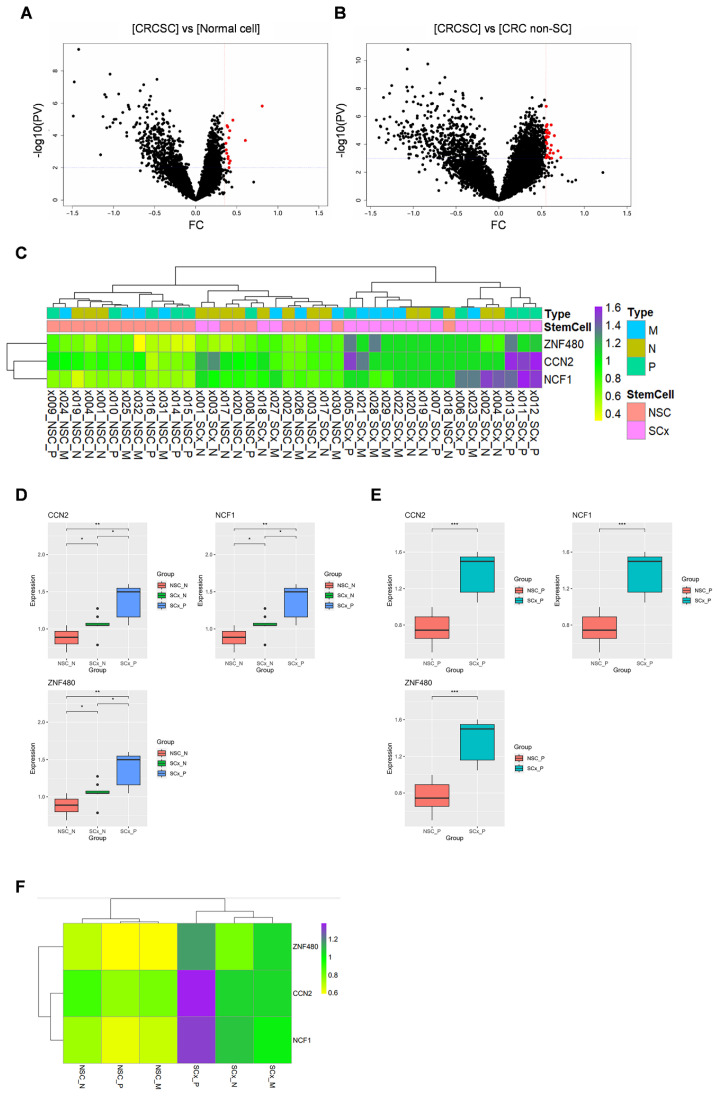
Visualization of differentially expressed genes (DEGs) between colorectal cancer (CRC) stem cells (CRCSCs) and normal cells, and between CRCSCs and CRC non-stem cells (CRC non-SCs). (**A**) DEGs between CRCSCs and normal cells were visualized using a volcano plot. Genes overexpressed in CRCSCs are highlighted in red based on the criteria of FC > 0.35 and *p*-value < 0.001. (**B**) DEGs between CRCSCs and CRC non-SCs are displayed in a volcano plot, with genes overexpressed in CRCSCs visualized in red, using a threshold of FC > 0.55 and *p*-value < 0.0001. (**C**) Expression levels of ZNF480, CCN2, and NCF1 across regions of interest (ROIs) were visualized using a heatmap. (**D**,**E**) Differences in expression of these three genes between normal cells and CRCSCs, and between CRCSCs and CRC non-SCs, are illustrated using box plots. (**F**) Expression levels of the three genes in CRCSCs, CRC non-SCs, CRC with liver metastasis stem cells, CRC with liver metastasis non-stem cells, normal stem cells, and normal non-stem cells were visualized with a heatmap. (* *p*-value < 0.05, ** *p*-value < 0.01 and *** *p*-value < 0.001).

We obtained expression data for 18,676 genes across 32 ROIs through GeoMx DSP analysis. DEGs between CRCSCs and normal colon cells (comprising both normal stem and non-stem cells) were visualized using a volcano plot. In CRCSCs, 15 genes were overexpressed (Figure 1A, depicted as red dots). Using a volcano plot, the DEGs between CRCSCs and CRC non-SC groups were visualized based on the following cutoff values: fold change >0.55 and *p*-value < 0.0001 (Figure 1B); 30 genes were overexpressed in CRCSCs. Three genes, *CCN2*, *NCF1*, and *ZNF480*, were identified in both groups. The expression levels of the three genes across each ROI were visualized using a heat map (Figure 1C). Differences in the expression of the three genes across groups are represented using boxplots. *CCN2*, *NCF1*, and *ZNF480* expression in the CRCSC group was significantly higher than that in both the normal colon non-stem cell and normal colon stem cell groups (Figure 1D). In addition, *CCN2*, *NCF1*, and *ZNF480* expression in the CRCSC group was significantly higher than that in the CRC non-SC group (Figure 1E). Average expression levels across the groups are depicted in a heatmap (Figure 1F). The findings indicate that *CCN2*, *NCF1*, and *ZNF480* are overexpressed in CRCSC.

PV, *p*-value; FC, fold change; CRCSC, colorectal cancer stem cell; CRC non-SC, colorectal cancer non-stem cell; x000, ROI number; NSC, non-stem cell; SCx, stem cell; P, primary cancer; M, CRC with liver metastasis cancer; N, normal colon.

### 2.2. Relationship Between CCN2 Expression and CRC Prognosis

**Figure 2:**
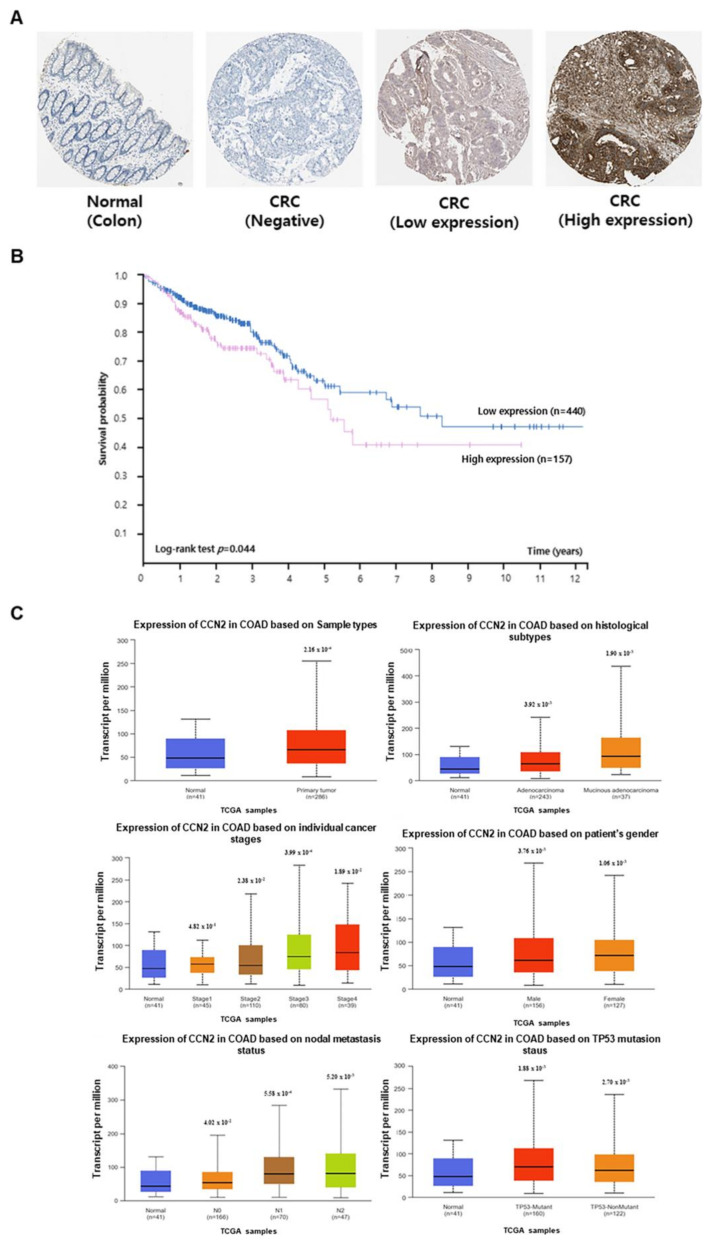
Association of *CCN2* expression in CRC tissues. (**A**) Verification of *CCN2* expression in normal colon tissue and CRC tissue from The Human Protein Atlas database. (**B**) The survival rate of CRC patients according to *CCN2* expression, determined using Kaplan–Meier analysis. (**C**) Using the UALCAN database, *CCN2* expression was examined across various clinicopathological characteristics, including tissue type, CRC stage, gender, nodal metastasis, and TP53 mutation.

*CCN2* expression data were obtained from The Human Protein Atlas and the UALCAN databases. CCN2 protein expression in normal and cancerous colon tissues was examined using The Human Protein Atlas database (Figure 2A). Based on *CCN2* expression, survival rates of patients with CRC were determined using Kaplan–Meier analysis and data from The Human Protein Atlas. Patients with high *CCN2* expression exhibited lower survival rates than those with low *CCN2* expression (Figure 2B).

Statistical analysis of data from the UALCAN database revealed that the mRNA expression of CCN2 was higher in colon cancer tissues than in normal colon tissues. *CCN2* expression was higher in adenocarcinoma and mucinous adenocarcinoma than in normal tissues and was more highly expressed in disease stages 3 and 4. Additionally, *CCN2* expression was higher in colorectal adenocarcinoma (COAD) tissues than in normal tissues across various clinicopathological features, including sex, nodal metastasis, and TP53 mutation status (Figure 2C).

### 2.3. RNA Expression in CRCs and Non-Stem Cells in CRCs That Metastasized to the Liver

**Figure 3:**
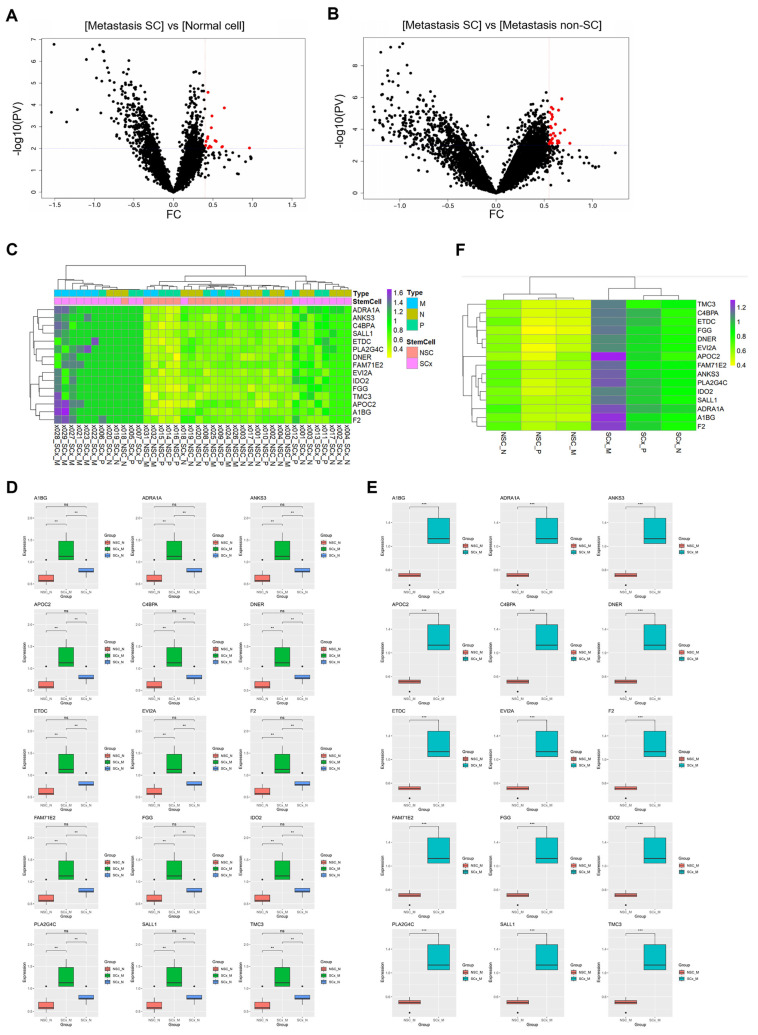
Visualization of differentially expressed genes (DEGs) between metastasis SCs and normal cells, and between metastasis SCs and metastasis non-SCs. (**A**) DEGs between metastatic SCs and normal cells were visualized using a volcano plot. Genes overexpressed in metastatic SCs are highlighted in red, using a criterion of FC > 0.4 and *p*-value < 0.001. (**B**) DEGs between metastatic SCs and Metastasis non-SCs were visualized in a volcano plot, highlighting genes with FC > 0.55 and *p*-value < 0.0001 in red. (**C**) Expression levels of 15 commonly overexpressed genes across regions of interest (ROIs) were visualized using a heatmap. (**D**,**E**) Differences in the expression of these 15 genes between normal cells and metastatic SCs, and between metastatic SCs and metastatic non-SCs, are illustrated using box plots. (**F**) Expression levels of the 15 genes in CRCSCs, CRC non-SCs, CRC with liver metastasis SCs, CRC with liver metastasis non-SCs, normal stem cells, and normal non-stem cells were visualized with a heatmap. (** *p*-value < 0.01 and *** *p*-value < 0.001).

Gene (n = 18,676) expression data were compared between the CRC with liver metastasis cancer stem cells (CRCs with liver metastasis SCs) group and the normal colon cells and the CRC with liver metastasis non-SC groups. DEGs between the CRC with liver metastasis SC group and normal colon cells (including both normal stem and non-stem cells) were identified with a fold change > 0.4 and a *p*-value < 0.001. In CRC with liver metastasis SCs, 15 genes were overexpressed (Figure 3A, represented as red dots). Thirty-three DEGs were identified between the CRC with liver metastasis SC group and the CRC with liver metastasis non-SC group (Figure 3B, based on the following criteria of fold change > 0.55 and *p*-value < 0.0001).

In the two comparison groups, 15 genes were commonly overexpressed in CRC SCs with liver metastasis, namely *A1BG*, *ADRA1A*, *ANKS3*, *APOC2*, *C4BPA*, *DNER*, *ETDC*, *EVI2A*, *F2*, *FAM71E2*, *FGG*, *IDO2*, *PLA2G4C*, *SALL1*, and *TMC3*. The expression of the 15 genes across each ROI was visualized using a heatmap (Figure 3C). To compare expression across groups (CRC with liver metastatic SCs, normal cells, and CRC with liver metastatic non-SCs), a boxplot was used (Figure 3D,E). Fifteen genes were specifically and significantly overexpressed in CRC with metastatic liver SCs. The average expression levels across the groups are depicted in a heatmap (Figure 3F). *APOC2* exhibited the highest expression levels in CRC with liver metastatic SCs.

PV, *p*-value; FC, fold change; Metastasis SC, CRC with liver metastasis stem cell; Metastasis non-SC, CRC with liver metastasis non-stem cell; x000, ROI number; NSC, non-stem cell; SCx, stem cell; P, primary cancer; M, CRC with liver metastasis cancer; N, normal colon.

### 2.4. Association Between APOC2 Expression and CRC Prognosis

**Figure 4:**
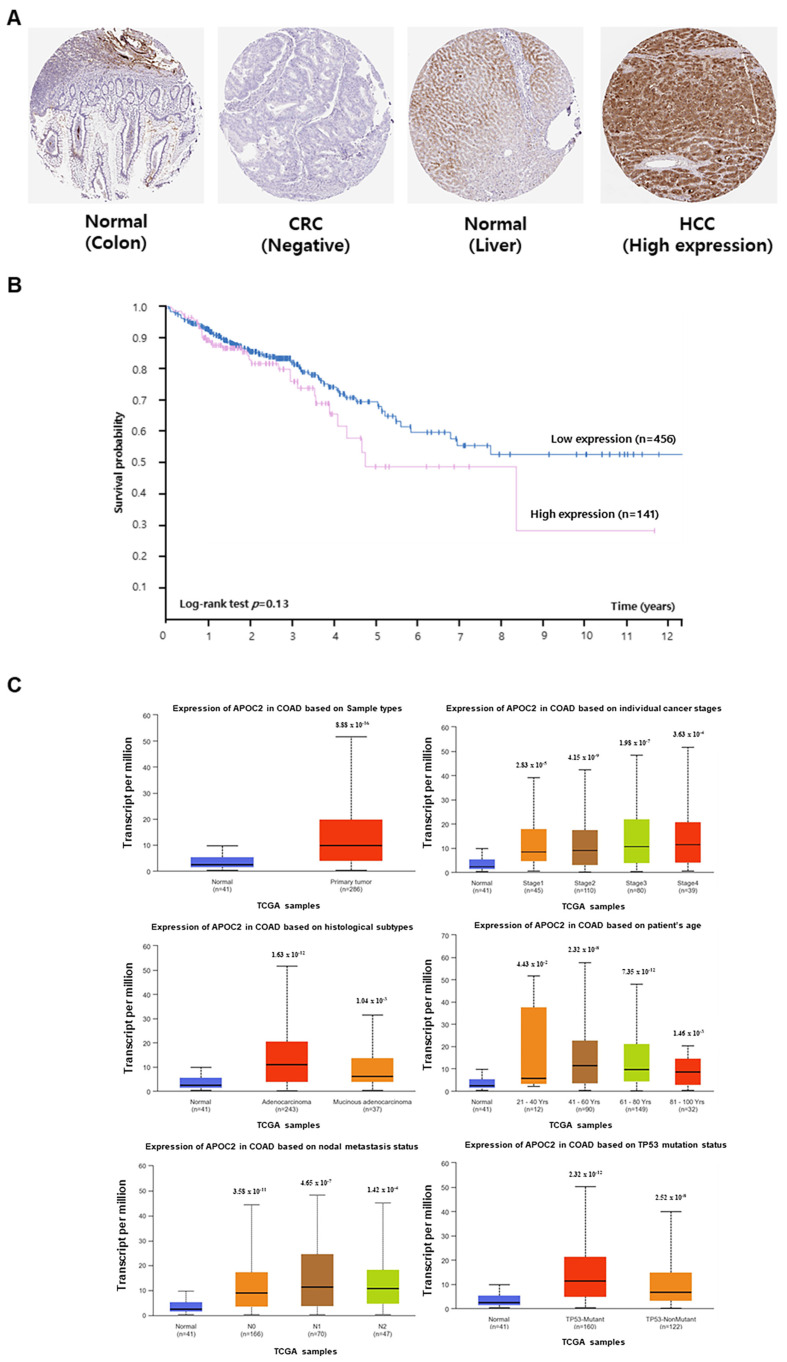
Verification of *APOC2* gene expression in tissues. (**A**) Expression of *APOC2* in normal colon tissue, CRC tissue, and hepatocellular carcinoma was confirmed using The Human Protein Atlas. (**B**) The survival rate of CRC patients based on *APOC2* expression levels was determined through Kaplan–Meier analysis. (**C**) Expression of *APOC2* across various clinicopathological characteristics, including tissue type, CRC stage, gender, nodal metastasis, and TP53 mutation status, was examined using data from the UALCAN database.

The association between *APOC2* expression and the prognosis of patients with CRC was investigated using data from The Human Protein Atlas and UALCAN databases. APOC2 protein expression was confirmed in normal colon, CRC, and hepatocellular carcinoma tissues using The Human Protein Atlas database (Figure 4A). Although the high APOC2 expression group showed a trend toward lower survival compared to the low expression group, this difference was not statistically significant (log-rank test, *p* = 0.13) (Figure 4B).

Statistical analysis of data from the UALCAN database revealed that APOC2 RNA expression was higher in CRC tissues. Furthermore, in COAD tissues, *APOC2* expression was higher than that in normal tissues across individual cancer stages, sample types, ages, nodal metastasis, and TP53 mutation status (Figure 4C).

### 2.5. Confirming CSC Association Through Functional Enrichment Analysis of DEGs

**Figure 5:**
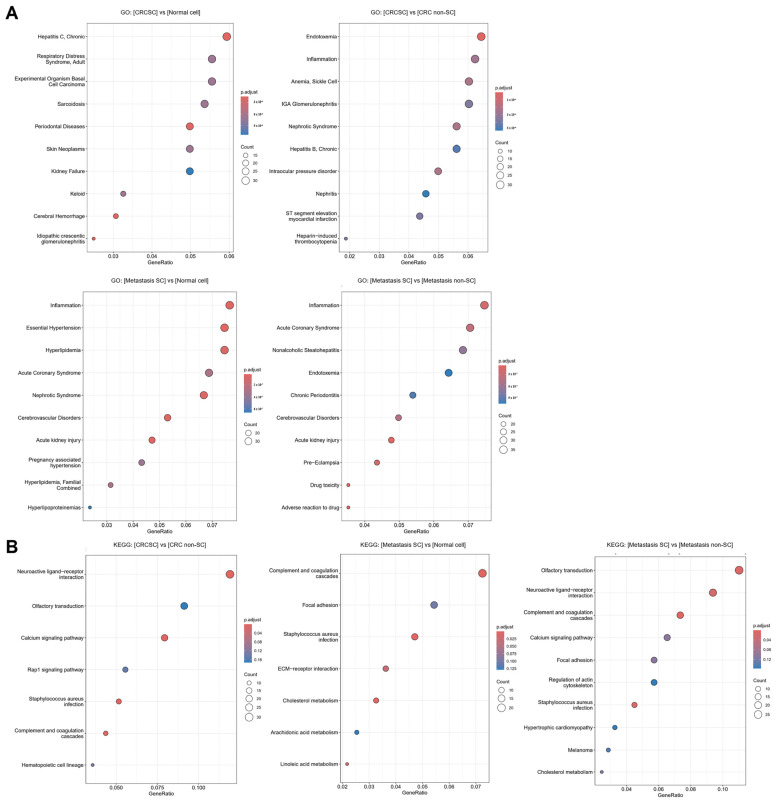
Enrichment analysis using Gene Ontology (GO) and Kyoto Encyclopedia of Genes and Genomes (KEGG) for differentially expressed genes (DEGs). (**A**) GO analysis was utilized to visualize the DEGs for each group with a bubble plot, revealing 10 enrichment terms per group. (**B**) KEGG analysis for the DEGs of each group identified 7 to 10 enrichment terms per group, which were visualized using bubble plots.

Enrichment analysis of DEGs across groups was conducted using GO and KEGG. In the GO analysis, ten enrichment terms were identified for each comparison group and visualized using a bubble plot (Figure 5A). In the CRCSCs and CRC with liver metastasis SC groups, terms related to inflammation, stem cell differentiation, and self-renewal were identified. After conducting KEGG analysis, seven enrichment terms were identified for each of the comparisons: between CRCSCs and CRC non-SCs, and between CRC with liver metastasis SCs and normal colon cells. In the analysis of the CRC with liver metastasis SCs and CRC with liver metastasis non-SC groups, ten enrichment terms were identified (Figure 5B). The terms identified through KEGG analysis were found to be associated with pathways related to cancer cell growth, migration, and metastasis, as well as stem cell differentiation and self-renewal.

### 2.6. Functional Role of CCN2 in Colorectal Cancer Cells

**Figure 6:**
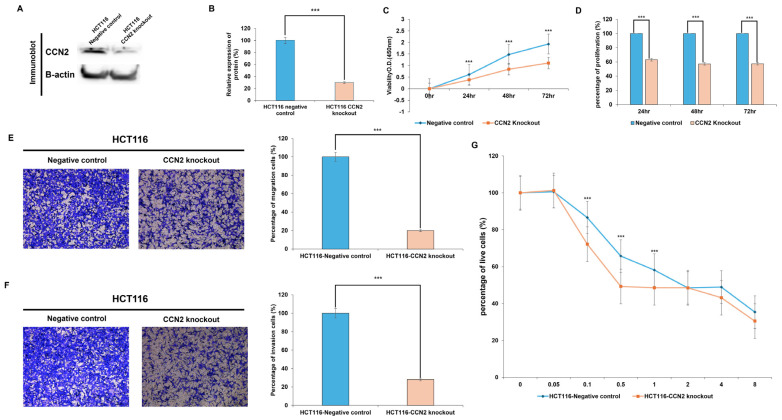
Functional role of CCN2 in colorectal cancer cells. (**A**,**B**) Western blot analysis showing efficient CCN2 knockout in HCT116 cells using the CRISPR-Cas9 system. Quantification demonstrated ~70% reduction in CCN2 protein levels compared with the negative control (*** *p* < 0.001). (**C**,**D**) WST-1 proliferation assay of CCN2-knockout and control HCT116 cells at 24, 48, and 72 h. CCN2 knockout markedly suppressed cell proliferation compared with controls (*** *p* < 0.001). (**E**) Representative images and quantification of Transwell migration assays. CCN2-knockout cells displayed an ~80% decrease in migration relative to controls (*** *p* < 0.001). (**F**) Representative images and quantification of Transwell invasion assays. CCN2-knockout cells exhibited an ~72% reduction in invasion compared with controls (*** *p* < 0.001). (**G**) Dose–response curves showing oxaliplatin sensitivity. CCN2-knockout cells were significantly more sensitive to low–moderate concentrations of oxaliplatin (0.1–1 μg/mL) compared with controls (*** *p* < 0.001), while differences diminished at higher concentrations. Data are presented as mean ± SD from three independent experiments. Statistical significance was determined using Student’s *t*-test (*** *p* < 0.001).

To investigate the functional role of CCN2 in colorectal cancer, CCN2-knockout HCT116 cells were generated using the CRISPR-Cas9 system. Western blot analysis confirmed a significant reduction in CCN2 protein expression (~70%) in knockout cells compared with negative controls (*p* < 0.001) (Figure 6A,B).

Functional assays revealed that CCN2 depletion markedly suppressed tumorigenic properties. In proliferation assays, CCN2-knockout cells displayed significantly lower viability compared with controls at 24 h (−37%, *p* < 0.001), 48 h (−43%, *p* < 0.001), and 72 h (−43%, *p* < 0.001) (Figure 6C,D).

Similarly, cell motility was impaired by CCN2 loss. In the migration assay, knockout cells exhibited an ~80% reduction in migratory capacity, with only 19.8% of cells migrating relative to controls (*p* < 0.001) (Figure 6E). In the invasion assay, the invasive capacity was reduced by ~72%, with only 28% of invading cells compared with controls (*p* < 0.001) (Figure 6F).

Finally, the role of CCN2 in drug resistance was examined by treating cells with oxaliplatin. CCN2-knockout cells exhibited significantly greater sensitivity at low to moderate oxaliplatin concentrations (0.1 μg/mL: −14%, *p* < 0.001; 0.5 μg/mL: −16%, *p* < 0.001; 1 μg/mL: −10%, *p* < 0.001) compared with controls. No significant differences were observed at higher concentrations (2–8 μg/mL), where both groups showed similar viability (Figure 6G).

Collectively, these results demonstrate that CCN2 promotes proliferation, migration, invasion, and oxaliplatin resistance in colorectal cancer cells, highlighting its potential as a therapeutic target.

### 2.7. Association Between CCN2 Expression and Cancer Stem Cell Markers in Colorectal Cancer

**Figure 7:**
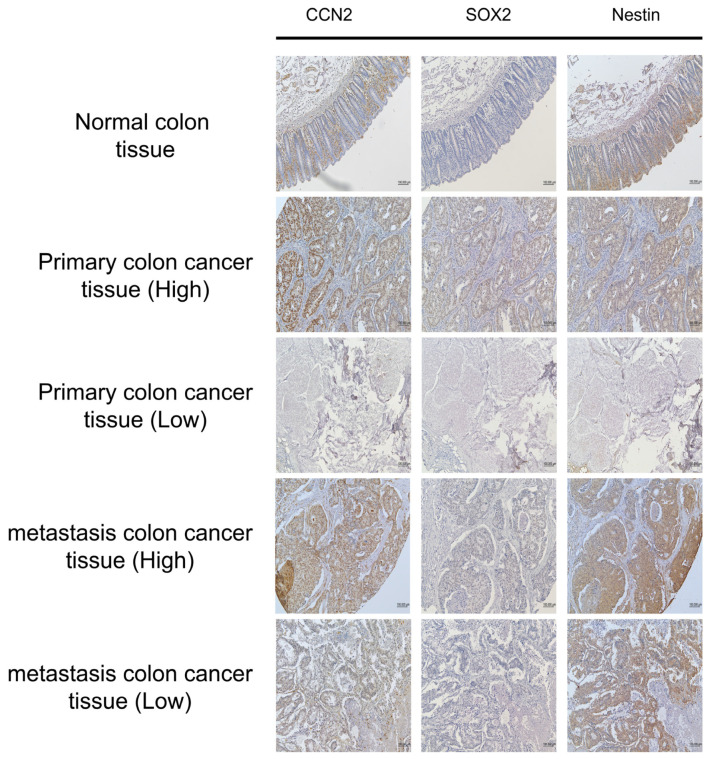
Immunohistochemical expression of CCN2, SOX2, and Nestin in colorectal cancer tissues. Immunohistochemical staining of CCN2, SOX2, and Nestin in normal colon, primary colorectal cancer, and metastatic colorectal cancer tissues. Representative images are shown (magnification ×100).

Immunohistochemistry (IHC) was performed on tissue microarray (TMA) slides comprising normal colon (n = 10), primary CRC (n = 40), and metastatic CRC (n = 10) samples to evaluate the correlation between CCN2 expression and the CSC markers SOX2 and Nestin (Nes) (Figure 7). Consecutive TMA slides from the same tissue blocks enabled direct comparison of expression patterns within identical histological contexts.

All normal colon tissues were negative for CCN2, SOX2, and Nestin expression. Among 50 CRC cases (primary and metastatic), SOX2 and Nestin expressions were stratified according to CCN2 status (Table 1). In cases with low CCN2 expression (n = 32), SOX2 was low in 21 cases and high in 11 cases, whereas Nestin was low in 15 cases and high in 17 cases. In contrast, all 18 cases with high CCN2 expression exhibited high SOX2 and Nestin expression.

These findings demonstrate a strong positive association between CCN2 expression and the expression of CSC markers SOX2 and Nestin in colorectal cancer tissues.

## 3. Discussion

Colorectal cancer (CRC) patients with distant metastasis experience a drastic decline in survival, with particularly high mortality associated with liver and peritoneal involvement [3,4]. Increasing evidence indicates that cancer stem cells (CSCs) are critical drivers of CRC initiation, progression, recurrence, and metastasis [11]. In this study, spatial transcriptomic profiling of normal colon, primary CRC, and liver metastatic tissues revealed distinct CSC-associated gene signatures. CCN2, NCF1, and ZNF480 were highly expressed in CSCs from primary CRC tissues, while APOC2, together with A1BG, ADRA1A, ANKS3, C4BPA, DNER, ETDC, EVI2A, F2, FAM71E2, FGG, IDO2, PLA2G4C, SALL1, and TMC3, was predominantly upregulated in liver-metastatic CSCs. Among these, CCN2 and APOC2 were the most robustly expressed genes in their respective contexts, with expression levels higher in COAD tissues than in normal colon tissues across various clinicopathological features.

CCN2, also known as connective tissue growth factor, regulates proliferation, differentiation, and migration in normal physiology and has been implicated in cancer cell proliferation, adhesion, drug resistance, and metastasis [21]. CCN proteins are also linked to key stem cell–related signaling pathways, including Wnt, BMP, and TGF-β/Smad [22,23,24,25], supporting their role in sustaining CSC functions. In our study, IHC analysis of tissue microarrays revealed that CCN2 expression was strongly correlated with CSC markers SOX2 and Nestin, further supporting its association with stemness. Functionally, CCN2 knockout in HCT116 cells significantly suppressed proliferation, migration, and invasion, and enhanced sensitivity to oxaliplatin at low-to-moderate concentrations, confirming its role in promoting tumorigenicity and chemoresistance in CRC cells.

APOC2, a regulator of fatty acid transport and lipid metabolism, has been associated with tumor formation, invasion, and metastasis, and its high expression correlates with poor prognosis in CRC [26]. Previous studies in glioblastoma have shown that CSCs contain lower lipid levels than non-CSCs, with lipid depletion enhancing stem-like characteristics [27]. These findings suggest that APOC2 may promote stemness in liver-metastatic CSCs by facilitating lipid degradation and metabolic reprogramming.

Although the spatial transcriptomic analysis in this study was based on tissue samples from only two patients, which is a clear limitation due to the small sample size, we addressed this issue by integrating TCGA data to evaluate CCN2 and APOC2 expression and their clinicopathological characteristics in a larger cohort of colorectal cancer patients.

Furthermore, to strengthen the association between CCN2 expression and cancer stemness, we performed functional assays using colorectal cancer cell lines to assess stem cell properties and tumorigenic potential according to CCN2 expression levels. In addition, we utilized the same TMA slide used for the spatial transcriptomic analysis to examine the correlation between CCN2 expression and CSC markers in a broader patient cohort. These complementary approaches help to compensate for the limited number of spatial transcriptomic samples and provide more robust evidence supporting the role of CCN2 and APOC2 in CRC progression and stemness.

Together, our findings identify CCN2 and APOC2 as critical CSC-associated genes in CRC. CCN2 enhances proliferation, invasiveness, and resistance in primary CRC cells, while APOC2 may drive stemness and metastatic adaptation in liver lesions. Additional studies are warranted to elucidate the molecular mechanisms underlying these effects and to explore the functional roles of other candidate genes (NCF1, ZNF480, A1BG, ADRA1A, ANKS3, C4BPA, DNER, ETDC, EVI2A, F2, FAM71E2, FGG, IDO2, PLA2G4C, SALL1, and TMC3). Ultimately, CCN2 and APOC2 may serve as promising biomarkers and therapeutic targets for CRC stem cells, with the potential to improve patient outcomes by overcoming recurrence and metastasis.

## 4. Materials and Methods

### 4.1. Case Selection

The tissue microarray (TMA) slide was purchased from SuperBioChips (Seoul, Republic of Korea, https://www.tissue-array.com/, accessed on 9 August 2021) and constructed using tissues from 40 patients diagnosed with CRC. The TMA slide comprised 59 samples, including 40 cores of CRC (primary cancer), 10 cores of CRC with liver metastasis, and nine cores of normal colon tissue (adjacent to cancer). We selected samples for digital spatial profiling from patients with colorectal cancer (CRC) who had matched normal colon tissue, primary CRC tissue, and CRC tissue with liver metastasis. As a result, two patients were selected. Core samples from these two patients were extracted from the tissue microarray (TMA) slide. Both patients were diagnosed with stage IVA adenocarcinoma. Clinical information was provided by SuperBioChips and is presented in Table 2.

For the immunohistochemistry (IHC) analysis to evaluate the association between CCN2 expression and cancer stem cell markers, the same TMA slide was used. Statistical analyses were performed on 50 cores, excluding normal colon tissue, out of a total of 59 cores on the TMA slide.

### 4.2. GEOMX Digital Spatial Profiling

RNA profiling was performed on TMA slides using NanoString GeoMx DSP (NanoString Technologies, Seattle, WA, USA). We obtained eight consecutive 4-μm-thick sections of FFPE tissues (four for QC validation and four for RNA analysis). For hybridization, tissue sections were affixed to positively charged Leica Bond Plus slides (Cat# S21.2113. A), according to the manufacturer’s protocol [28]. Immunohistochemical analysis was performed using the Leica Bond Rx autostainer system (Leica Biosystems, Nussloch, Germany) that includes deparaffinization and antigen retrieval. The tissues were then treated with a cocktail of antibodies constituting the immunofluorescent biomarkers. These immunofluorescent biomarkers were used as visualization markers, and SYTO13 (GeoMx Solid Tumor TME Morphology Kit, Cat# 121300301) was utilized as a DNA marker [19,29]. To identify tumors and CSCs, CD44 (GeoMx TAP antibody morphology marker, Cat# NB500-481 AF647, Seattle, WA, USA) and EpCAM (GeoMx TAP antibody morphology marker, cat # ab213500) staining was performed. EpCAM+/CD44- cells were used as markers to identify non-CRCSCs, and EpCAM+/CD44+ cells were used as markers to identify CRCSCs.

### 4.3. Selection of Regions of Interest (ROIs)

**Figure 8:**
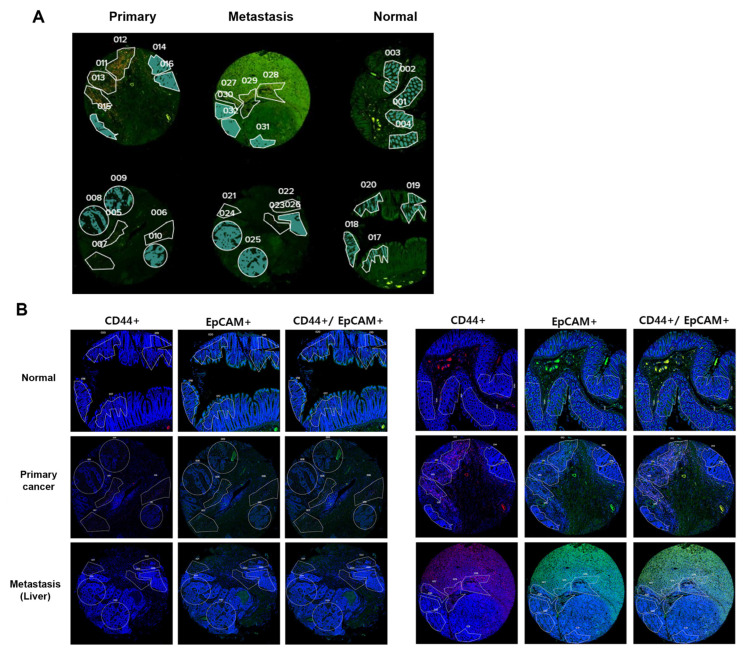
Region of interest (ROI) selection based on CD44 and EpCAM expression in colorectal cancer (CRC) patient tissue samples. (**A**) A total of 32 ROIs were selected from CRC tissue, CRC with liver metastasis tissue, and normal colon tissue obtained from two CRC patients. (**B**) Areas expressing stem cell marker, CD44, and colorectal epithelial cell marker, EpCAM, are designated as ROIs based on their expression profiles: areas with EpCAM+/CD44- and areas with EpCAM+/CD44+.

GeoMx software (27.12.2022.) was used to define ROIs. ROIs larger than 1000 μm^2^ were selected by a pathologist using segmentation and circle selection tools. A total of 32 ROIs were designated, including normal cores (4 ROIs), primary cores (6 ROIs), and metastatic cores (6 ROIs). The RNA within the ROIs was captured and profiled using the GeoMx Human Whole Transcriptome Atlas (NanoString, Seattle, WA, USA), which allows for the detection of approximately 18,000 RNA targets [30]. The ROIs were based on CD44 expression. Each ROI was subdivided into areas of interest (AOIs) within the colon epithelium (EpCAM positive), classifying the CD44 positive sections as CSCs and the CD44 negative sections as cancer cells or normal colon epithelial cells. For the DSP RNA expression evaluation, 40 AOIs were selected (non-stem cell 20 AOIs, stem cell 20 AOIs) (Figure 8). The defined AOIs were individually illuminated using the ultraviolet light of the GeoMx DSP device, releasing oligonucleotide tags bound to the antibodies present in each AOI. The released tags were quantified using nCounter, and the counts were re-mapped to the tissue location, creating a spatially resolved digital profile of analyte abundance. Digital counts were normalized to those of the housekeeping genes [31].

### 4.4. Data Analysis

Gene expression levels were determined using a NanoString DSP assay. Differentially expressed genes (DEGs) were selected using the following cut-off values: fold changes and *p*-values. Fold changes and *p*-values were retrieved using the default “t.test” in R. The total fold change and *p*-values were visualized as volcano plots, and heatmaps were plotted using the “pheatmap” R package. In addition, the R package “ggplot2” was used to generate boxplots. Gene ontology (GO) analysis was performed using the “DOSE” R package with the “enrichDGN” function. Kyoto Encyclopedia of Genes and Genomes (KEGG) analysis was performed by “clusterProfiler” R package with “enrichKEGG” function. KEGG pathways were visualized using the “pathview” R package. Enrichment terms were obtained, and their associated networks were visualized using the “DOSE” R package with the “enrichDGN” and “cnetplot” functions, respectively.

### 4.5. Expression Analysis

CCN2 and APOC2 expression in normal and tumor tissues was analyzed using The Human Protein Atlas (https://www.proteinatlas.org/) and The University of Alabama at Birmingham CANcer data analysis portal (UALCAN) databases (http://ualcan.path.uab.edu/index.html accessed on 9 August 2021). Additionally, the CCN2 and APOC2 gene expression patterns in CRC were analyzed using the UALCAN database, and the association of gene expression with clinical and pathological factors, such as CRC subtypes, sex, metastasis, and TP53 mutations, was evaluated.

### 4.6. Gene Knockout by CRISPR-Cas9 System

The CRISPR-Cas9 system was used to generate a CCN2 knockout in HCT116 colorectal cancer cells. The sequence information for the CCN2-targeting single guide RNA (sgRNA; Thermo Fisher Scientific, Waltham, MA, USA) is 5′-CACCGTACCACCGAAGATGC- 3′. HCT116 cells were thawed and seeded at a density of 1 × 10^4^ cells per well in 24-well plates within four passages after thawing. Cells were cultured in RPMI 1640 medium supplemented with 10% fetal bovine serum (FBS; Thermo Fisher Scientific) at 37 °C in a humidified 5% CO_2_ incubator for 24 h. When the cells reached approximately 50–60% confluence, a CRISPR-Cas9 RNP complex was prepared by combining 7.5 pmol of Cas9 Protein v2 (Thermo Fisher Scientific), 7.5 pmol of sgRNA, Lipofectamine Cas9 PLUS reagent, and Lipofectamine CRISPRMAX reagent (Thermo Fisher Scientific) according to the manufacturer’s instructions. The mixture was added to each well, and the cells were incubated for 48 h under standard culture conditions (37 °C, 5% CO_2_). Following transfection, the medium was removed by gentle aspiration and replaced with fresh complete medium. After an additional 24 h recovery period, the cells were seeded at a density of 1 cell per well in 96-well plates for clonal selection. Individual colonies were allowed to grow and expand separately. Immunoblot analysis was performed to confirm CCN2 knockout, and clones with complete loss of CCN2 expression were selected for further experiments.

### 4.7. Immunoblot Analysis

For protein extraction, CCN2 knockout and control HCT116 cells (1 × 10^7^) were lysed in 400 μL PRO-PREP™ protein extraction solution (iNtRON Biotechnology, Seongnam-si, Gyeonggi-do, Republic of Korea). Lysates were incubated at −20 °C for 1 h and centrifuged at 12,000× *g* for 20 min at 4 °C. Protein concentrations were determined using the Pierce™ BCA Protein Assay Kit (Thermo Fisher Scientific) with bovine serum albumin standards (0–2000 μg/mL). Absorbance was measured at 562 nm using a Multiskan™ GO microplate reader (Thermo Fisher Scientific).

Equal amounts of protein (30 μg) were mixed with 2× Laemmli buffer (Bio-Rad, Hercules, CA, USA), boiled for 15 min at 100 °C, and separated by SDS-PAGE. Proteins were transferred onto PVDF membranes (Immobilon, Millipore, Burlington, MA, USA), blocked with 5% skim milk for 1 h, and incubated overnight at 4 °C with anti-CCN2 polyclonal antibody (1:1000; Proteintech, Rosemont, IL, USA) in 5% BSA. After washing, membranes were incubated with HRP-conjugated secondary antibody for 4 h at room temperature, and signals were detected using ECL reagents (Advansta, San Jose, CA, USA).

### 4.8. Immunofluorescence

Tissue microarray (TMA) slides were obtained from SUPERBIO CHIPS (Seoul, Republic of Korea), containing colorectal cancer tissues (n = 40), normal colon tissues (n = 10), and metastatic carcinoma samples (n = 10). Clinical data, including age, sex, pathological stage (pT, pN, overall stage), metastasis status, and survival information, were available for each case.

Slides were deparaffinized in xylene (three times) and rehydrated through graded ethanol (100%, 95%, 80%, 70%; 3 min each). Antigen retrieval was performed in citrate buffer. After blocking with 1% BSA, tissue sections were incubated with primary antibodies overnight at 4 °C in a humidified chamber, followed by incubation with secondary antibodies (1% BSA) for 4 h at room temperature. Immunoreactivity was visualized using diaminobenzidine (DAB; DAKO, Glostrup, Denmark), and nuclei were counterstained with hematoxylin.

### 4.9. Cell Proliferation Assay

Cell proliferation was evaluated using the WST-1 assay with the Cyto X reagent (LPS Solution, Seoul, Republic of Korea). Transfected and control HCT116 cells were seeded at a density of 1 × 10^4^ cells/well in 96-well plates. At 24 h intervals, Cyto X reagent was added, and cells were incubated for 1 h at 37 °C. Absorbance was measured at 450 nm using a Multiskan™ GO microplate reader (Thermo Fisher Scientific, Waltham, MA, USA).

### 4.10. Migration and Invasion Assay

Cell migration and invasion were assessed using 6.5 mm Transwell inserts with 8 μm pores (Corning Inc., Corning, NY, USA). For invasion assays, the upper chamber was pre-coated with 50 μL Matrigel (BD Biosciences, San Jose, CA, USA). Transfected and control cells (2 × 10^5^) were seeded in 200 μL serum-free medium in the upper chamber, and the lower chamber was filled with 750 μL RPMI 1640 medium containing 10% FBS and 1% penicillin–streptomycin. After 48 h incubation at 37 °C in a 5% CO_2_ atmosphere, migrated or invaded cells on the lower membrane surface were fixed with 4% paraformaldehyde and stained with 0.05% crystal violet.

### 4.11. Oxaliplatin Resistance

Oxaliplatin resistance was assessed by measuring cell viability using the Cyto X reagent (LPS Solution, Seoul, Republic of Korea). CCN2 knockout and control HCT116 cells (1 × 10^4^ cells/well) were seeded in 96-well plates and incubated for 24 h. Cells were then treated with oxaliplatin (Sigma-Aldrich, Burlington, MA, USA), dissolved in 5% glucose solution, at final concentrations of 0–8 μg/mL (0, 0.05, 0.1, 0.5, 1, 2, 4, and 8 μg/mL). After 48 h incubation at 37 °C, Cyto X reagent (diluted in serum-free medium) was added to each well and incubated for 1 h. Absorbance was measured at 450 nm using a Multiskan™ GO microplate reader (Thermo Fisher Scientific, Waltham, MA, USA).

## Figures and Tables

**Table 1:** Correlation of CCN2 expression with SOX2 and NES in colorectal cancer patients.

	**CCN2 Expression**		**Total**	***p*-Value**
	Low (N = 32)	High (N = 18)	(N = 50)	
SOX2 Expression, N (%)				0.0001
LOW	21 (100)	0	21	
High	11 (37.9)	18 (62.1)	29	
Nestin Expression, N (%)				0.001
Low	15 (100)	0	15	
High	17 (48.6)	18 (51.4)	35	

**Table 2:** Clinical information.

No.	Age	Sex	Organ	Diagnosis	pTNM	Stage
Sample 1	53	M	Normal: Sigmoid colon Primary: Ascending colon Metastasis: Liver	Adenocarcinoma, poorly differentiated	T3N1bM1a	IVA
Sample 2	69	M	Normal: Ascending colon Primary: Cecum Metastasis: Liver	Adenocarcinoma, moderately differentiated	T2N1bM1a	IVA

## Data Availability

The datasets used and/or analyzed in the current study are available from the corresponding author upon reasonable request. The data discussed in this publication have been deposited in NCBI’s Gene Expression Omnibus (Edgar et al., 2002) [32] and are accessible through GEO Series accession number GSE271285 (https://www.ncbi.nlm.nih.gov/geo/query/acc.cgi?acc=GSE271285 accessed on 29 October 2025). To access the data before the publication of the paper, please visit the link and enter the token klyluimcfrapvub in the box.

## Abbreviations

CRC, colorectal cancer; mCRC, metastatic CRC; LV, leucovorin; CSCs, cancer stem cells; CRCSCs, CRC stem cells; FFPE, formalin-fixed paraffin-embedded; DSP, Digital Spatial Profiler; TMA, tissue microarray; AOIs, areas of interest; GO, Gene ontology; ROIs, regions of interest; KEGG, Kyoto Encyclopedia of Genes and Genomes; COAD, colorectal adenocarcinoma
